# Preserved glycemic control and baroreflex efficacy in young adult hypertensive female obese Zucker rats

**DOI:** 10.1152/ajpregu.00341.2020

**Published:** 2021-05-12

**Authors:** Parul Chaudhary, Paromita Das-Earl, Ann M. Schreihofer

**Affiliations:** Department of Physiology and Anatomy, University of North Texas Health Science Center, Fort Worth, Texas

**Keywords:** glucose tolerance, hyperglycemia, insulin resistance, metabolic syndrome, telemetry

## Abstract

Obese Zucker rats (OZRs) develop hypertension and hyperinsulinemia by 3 mo of age. Male OZRs also have diminished baroreflex-mediated activation of nucleus tractus solitarius (NTS) and bradycardia, which are improved by correcting their hyperglycemia. Conversely, 3-mo-old female OZRs and lean Zucker rats (LZRs) have equivalent baroreflex-mediated bradycardia that is impaired in 6-mo-old OZRs. We hypothesized that 3-mo-old female OZRs maintain NTS activation and baroreflexes coincident with glycemic control. We also hypothesized that 6-mo-old female OZRs develop impaired baroreflexes with hyperglycemia and diminished NTS activation. In 12- to 16-wk-old females, sympathetic nerve activity (SNA) and arterial pressure (AP) were higher in OZRs than LZRs. However, baroreflex-mediated inhibition of SNA and bradycardia were equivalent in female OZRs and LZRs. Unlike deficits in male OZRs, female OZRs and LZRs had no differences in phenylephrine-induced c-Fos expression in NTS or decreases in SNA and AP evoked by glutamate into NTS. Compared with hyperglycemia in male OZRs (217.9 ± 34.4 mg/dL), female OZRs had normal fed blood glucose levels (108.2 ± 1.6 mg/dL in LZRs and 113.6 ± 3.5 mg/dL in OZRs) with emerging glucose intolerance. Conscious 24- to 27-wk-old female OZRs had impaired baroreflex-mediated bradycardia, but fed blood glucose was modestly elevated (124.2 ± 5.2 mg/dL) and phenylephrine-induced c-Fos expression in NTS was comparable to LZRs. These data suggest that better glycemic control in 3-mo-old female OZRs prevents diminished NTS activation and baroreflexes, supporting the notion that hyperglycemia impairs these responses in male OZRs. However, 6-mo-old female OZRs had impaired baroreflex efficacy without diminished NTS activation or pronounced hyperglycemia, suggesting baroreflex deficits develop by different mechanisms in female and male OZRs.

## INTRODUCTION

With the rising global epidemic of obesity, the prevalence of metabolic syndrome (MetS) has grown significantly (1). Almost one-quarter of the world’s population exhibits signs of MetS (2), which comprises a cluster of traits that greatly increases the risk of cardiovascular disease and premature morbidity and mortality (3). Hypertension is the only recognized cardiovascular trait of MetS that is routinely measured and managed in the clinic (2), but short-term control of arterial pressure (AP) by baroreflexes is also impaired in people with obesity and MetS (4). Diminished baroreflexes promote increased variability in AP that can produce excursions of AP to dangerously high levels in people and animals with obesity (5–7). In the absence of hypertension, increased AP variability increases risk for organ damage, stroke, and cognitive decline (7–10). Reducing risks from impaired short-term control of AP requires a better understanding of mechanisms underlying the development of impaired baroreflexes in settings such as MetS.

Multiple attributes of MetS are individually associated with baroreflex efficacy and AP variability (4), namely obesity, hypertension, dyslipidemia, insulin resistance, and hyperglycemia (6, 11–16). These attributes of MetS also exacerbate each other, which contributes to their comorbid presentation. Insulin resistance promotes the development and progression of hypertension with MetS (17), and in patients with obesity, hypertension is associated with augmented impairment of baroreflexes (11). In the absence of obesity and hypertension, insulin resistance is also independently associated with impaired baroreflexes (15, 18). These interactions complicate the treatment of deleterious attributes of MetS and necessitate the consideration of comorbidities to improve otherwise detrimental outcomes.

Sex differences have been observed in the development of MetS traits in terms of presence, onset, and degree. Many of the recognized signs of MetS appear earlier and in worse severity in males. Compared with women, a similar measure of obesity in men is often associated with more severe dyslipidemia, hyperinsulinemia, impairment of glycemic control, and hypertension (19). Elevated sympathetic nerve activity (SNA) to cardiovascular targets is reliably observed in men with obesity but is often absent in women with obesity (20–24). Nevertheless, in men and women with obesity, SNA is consistently and positively correlated with AP (22, 23). Sex differences have been reported for baroreflex efficacy, even in healthy adults. Young women exhibit greater baroreflex efficacy compared with age-matched men (25) and postmenopausal women (26), and estrogen replacement in postmenopausal women improves baroreflex efficacy (27). Estrogen also protects against the development of insulin resistance and diabetes (28–30), so preservation of glycemic control may also contribute to the protection of baroreflexes in women with obesity. However, the influence of sex-based differences upon baroreflex efficacy and glucose homeostasis in the setting of obesity have not been elucidated.

Obese Zucker rats (OZRs) provide an excellent model for examining the development and interactions of MetS traits seen in humans with obesity. Owing to a mutation that produces dysfunctional leptin receptors, OZRs overeat standard rat chow and become obese due to hyperphagia and higher energetic efficiency (31, 32). Traits of MetS emerge after excess weight gain, and deleterious changes in autonomic regulation of cardiovascular function develop in adulthood (5, 33–35). In addition, OZRs have sex differences in the onset of impaired baroreflex-mediated changes in heart rate (HR) (5, 33, 36). Male OZRs develop hypertension and impaired baroreflex efficacy by 3 mo of age (5, 33). These traits occur with diminished activation of the nucleus tractus solitarius (NTS) by baroreceptor afferent nerves and impaired NTS-mediated changes in SNA and HR (33, 37). At this age, male OZRs have insulin resistance characterized by hyperinsulinemia and glucose intolerance, but fasting blood glucose is comparable in male OZRs and lean Zucker rats (LZRs) (38). However, when these male OZRs have access to food, blood glucose measured by telemetry is chronically and prominently elevated. Restoration of normal fed glucose levels in male OZRs enhances baroreflex-mediated activation of the NTS and bradycardia, even with the persistence of hyperinsulinemia and hypertension (38). These data suggest that in male OZRs, chronically elevated blood glucose contributes to impaired activation of the NTS and baroreflex efficacy. In contrast to male OZRs, at 3 mo of age, female OZRs have hypertension, but the magnitude of baroreflex-mediated bradycardia is comparable to female LZRs (36). However, by 6 mo of age, female OZRs have impaired baroreflex-mediated bradycardia (36), suggesting that female OZRs develop the same dysfunction with a delayed onset, as observed in humans with obesity (25, 26). The ability of female OZRs and LZRs to regulate blood glucose at either of these age ranges is not known.

We examined the hypotheses that preserved baroreflex bradycardia in 3-mo-old female OZRs is reflected in sympathetic baroreflexes and coincides with an ability to maintain glycemic control and baroreflex-mediated activation of the NTS. Blood glucose was measured continuously by telemetry in undisturbed rats with access to food, because fasting blood glucose levels are not adequate for detecting detrimental chronic hyperglycemia in male OZRs (38), and OZRs develop exaggerated rises in glucose with brief stressors (38, 39). Glucose tolerance was assessed because this measure is a strong indicator for prediabetes and risk of cardiovascular disease with MetS (3), and male OZRs develop glucose-related cardiovascular deficits before the onset of impaired fasting glucose and changes in HbA1c (35, 38). In addition, we examined the hypothesis that 6-mo-old female OZRs develop impaired baroreflex-mediated bradycardia coincident with a pronounced elevation in fed glucose levels and a decline in baroreflex-mediated activation of the NTS.

## MATERIALS AND METHODS

### Animals

Male and female OZRs [Lepr (fa/fa)] and LZRs [Lepr (+/+) and (+/fa)] from Charles River (Houston, TX) were individually housed in centralized animal care facilities kept at consistent humidity (60% ± 5%), temperature (24°C ± 1°C), and light cycle (lights on 7:00 AM to 7:00 PM). Rats were fed standard rat chow (Prolab RMH 1800, LabDiet). To correspond with the delayed development of impaired baroreflex bradycardia reported for 3- and 6-mo-old female OZRs (36), experiments were performed on young adult (12–17 wk) and older adult (24–27 wk), age-matched OZRs and LZRs. The University of North Texas Health Science Center Institutional Animal Care and Use Committee approved all animal experiments, which were performed in accordance with guidelines from the National Institutes of Health’s *Guide for Care and Use of Laboratory Animals* and the American Physiological Society’s *Guiding Principles for the Care and Use of Vertebrate Animals in Research and Training*.

### Surgical Preparation for Cardiovascular Measures in Conscious Rats

Young adult males and females (12- to 14-wk old) and older females (24- to 27-wk old) were anesthetized with isoflurane (5% in a ventilated box and then 2.0%–2.4% via a nose cone). A catheter was implanted into the left femoral artery to record AP and into the left femoral vein to infuse fluids. The free ends of the catheters were tunneled subcutaneously to exit between the scapulae (33). The rats were fitted with a tether and dual-channel swivel (Instech Laboratories) that was attached to a counterbalanced lever arm to allow them to move freely in a plexiglass cylindrical cage (MTANK/W and MTOP, Instech). The rats were allowed to recover after surgery for 24–48 h with access to food and water. To assess metabolic status in 24- to 27-wk-old conscious rats, a 0.5-mL blood sample was taken from the arterial line on the morning of the experiment, and the volume was replaced by flushing the line with isotonic saline.

### Baroreflex-Mediated Bradycardia and Activation of c-Fos Expression in the NTS in Conscious Rats

On the day of the experiment, each cylindrical cage was surrounded with a cover to minimize disturbance to the rat. To ensure similar environmental conditions, rats were infused in pairs (1 lean and 1 obese) separated by 30 min to allow for perfusion of each rat. The arterial line was connected through the swivel to a pressure transducer (NL108T2, Digitimer), and the venous line was connected through the swivel to an infusion pump (Model A-99, Razel). Baseline AP, mean AP, and HR were recorded for 30 min to ensure stability and then phenylephrine (PE) was infused (17–21 µL/min of 0.5 mg/mL in saline iv) to raise mean AP by ∼40 mmHg in 20–40 s. The baroreflex-mediated decrease in HR was measured during the first 5 min of the target rise in AP and then the rise in AP was maintained for 60–90 min to provide a consistent stimulus for c-Fos expression in the NTS. The mean AP was continuously monitored, and the rate of infusion was adjusted up or down (4–30 µL/min) to maintain the target AP. After 60 min, the PE-filled syringe was replaced with a saline-filled syringe, and the infusion continued with the same rate to slowly flush PE from the venous line and allow AP to return toward baseline by 90 min (33). Then, the rats were deeply anesthetized with urethane (1.5 g/kg iv bolus) for transcardial perfusion with 250 mL of phosphate-buffered saline (pH 7.4) followed by 500 mL of 4% phosphate-buffered paraformaldehyde (Electron Microscopy Sciences). The brains were extracted and stored in the same fixative for 48 h.

The 40-mmHg increase in AP was used to reliably produce a maximal baroreflex bradycardia and provide a consistent magnitude of rise in AP for robust and reliable of c-Fos expression in the NTS (33, 40). The fixed change in AP is essential, because the number of c-Fos+ neurons in the NTS varies by the magnitude of change in AP (40). Although the 40-mmHg rise could exceed the mean AP required to achieve a maximal decrease in HR, baroreceptor afferent nerves to the NTS could still be further activated within this range. In anesthetized male Zucker rats, SNA can plateau before aortic depressor nerve activity during infusion of PE (37). The rise in AP was maintained for 60 min to prevent a post-PE hypotensive stimulus that could affect c-Fos expression, particularly in OZRs. Although the maximum bradycardia is not maintained for the duration of the infusion, the persistence of bradycardia through the 60-min period suggests sustained activation of baroreceptor afferent nerves (33). Similar volumes of the PE solution are needed to sustain the 40-mmHg rise in AP despite impaired baroreflexes in male OZRs, perhaps due to their impaired mesenteric adrenergic vascular reactivity and equivalent blood volumes in age-matched OZRs and LZRs (33, 41). This volume and rate of infusion of saline produces no changes in mean AP and minimal c-Fos expression in the NTS (33, 40). The rats were perfused 90 min after the onset of the target AP, because PE-induced c-Fos expression peaks 1–2 h after the onset of the rise in AP (40).

### Immunohistochemistry for c-Fos Protein

Brain stems were sectioned with a Vibratome (30 µm, coronal plane) and stored at −20°C in a cryoprotectant solution (42). Immunohistochemistry for detection of c-Fos protein was performed on free-floating sections (every 1 in 6 sections) on an orbital shaker in solutions prepared in Tris-buffered saline (TBS, pH 7.4) at room temperature unless specified otherwise. Brain stem sections from OZRs and LZRs were processed together using the same solutions and timing to ensure consistent conditions between groups. The sections were incubated with 1% hydrogen peroxide (30 min) to block endogenous peroxidases, rinsed in TBS (3 times for 5 min), and blocked in 10% horse serum (45 min). Then, sections were incubated with a goat-anti c-Fos primary antibody (1:2,000; 4°C; 48 h; sc-52G, Santa Cruz Biotechnology), as previously described (33). After being rinsed in TBS, sections were incubated with a biotinylated donkey anti-goat secondary antibody (1:400; 1 h; 705-066-147, Jackson Laboratories) followed by an avidin-biotin solution (1 h; PK-6100; Vector Laboratories). Incubation with a nickel-intensified 3,3′-diaminobenzadine solution was used to reveal the c-Fos immunoreactivity. The reaction was carefully monitored for 8–10 min and terminated with a TBS rinse when staining became visible. The stained sections were mounted onto gelatin-coated slides and dried overnight. Slides were submerged through a series of alcohols and xylenes and then coated with DPX mounting medium (Sigma-Aldrich) to affix coverslips. The c-Fos-immunoreactive (c-Fos+) neurons were mapped and counted bilaterally in the NTS at four rostrocaudal levels using a Ludl-motor-driven stage and Neurolucida software (MicroBrightfield) (33).

### Surgical Preparation for Physiological Measures in Anesthetized Rats

Young adult Zucker rats were anesthetized with isoflurane (5% in a ventilated box and then 2.0%–2.4% via a nose cone) with 100% oxygen. After the adequacy of anesthesia was confirmed by toe pinch, femoral arterial and venous catheters were implanted. To assess metabolic status at this age range, a blood sample (0.5 mL) was taken from the arterial line, and the volume was replaced by flushing the line with sterile saline. After insertion of a tube into the trachea, rats were artificially ventilated. For LZRs, the ventilation rate was 55–65 strokes/min of 1 mL/100 g body weight (model 683; Harvard Apparatus). For OZRs, the initial ventilation was based on the tidal volume of the age-matched LZR, with further adjustment slightly upward to achieve an end-tidal CO_2_ comparable to the LZR (3.5%–4.0%; CapStar-100; CWE) at a similar rate of ventilation (37). Rats were placed in a stereotaxic instrument (David Kopf Instruments) with the bite bar set at −11 mm to facilitate exposure of the dorsal brain stem. The left greater splanchnic nerve was exposed retroperitoneally, isolated immediately distal to the adrenal branch, and placed on the bared tips of two Teflon-coated silver wires (A-M Systems). After carefully removing surrounding fluid, the nerve and exposed wires were encased in a silicone elastomer (Kwik-Sil; World Precision Instruments) (34, 37). To expose the dorsal surface of the brain stem, the occipital bone was exposed and removed and the underlying meninges were clipped and retracted. Rectal temperature was maintained at 37°C. After the surgical preparations were completed, isoflurane anesthesia was replaced by urethane (1.5 g/kg LZR body weight administered intravenously using 1.5 g/5 mL solution at 50 µL/min). After 30–45 min of recovery under urethane anesthesia, the rat was paralyzed with pancuronium (0.1 mL/100 g of a 1 mg/mL solution with 1/3 dose supplements/h; Hospira). To ensure no age differences between LZRs and OZRs, the experiments alternated between lean and obese rats.

### Assessment of Sympathetic Baroreflexes in Anesthetized Female Rats

To determine whether the absence of impaired baroreflex-mediated bradycardia in young adult female OZRs was due to equivalent changes in efferent nerve activity or compensations at the heart, we examined baroreflex-mediated changes in splanchnic SNA in female OZRs and LZRs. The sympathetic baroreflex was measured as previously reported in urethane-anesthetized male Zucker rats (5). To produce a steady rise in mean AP, PE was infused (50 µg/mL iv) until the splanchnic SNA reached a lower plateau within ∼2 min. After the mean AP and SNA had returned to within 90% of baseline, sodium nitroprusside was infused (50 µg/mL iv) to steadily reduce mean AP until SNA reached an upper plateau within ∼1 min. The rat was allowed to recover to baseline mean AP and SNA before receiving nanoinjections into the NTS.

### Nanoinjections into the NTS in Anesthetized Rats

Nanoinjections into the brain stem were performed using single-barrel glass pipettes pulled and cut to a 40- to 50-µm diameter tip that were fixed on a stereotaxic arm and connected to a pressure injection apparatus (Pressure System IIe; Toohey). The stereotaxic coordinates for the NTS were 0.5 mm lateral to the midline, 0.5 mm rostral to calamus scriptorius, and 0.5 mm ventral to the dorsal surface of the brain stem (33). To activate NTS neurons, glutamate (1 nmol in 50 nL) was prepared in artificial cerebrospinal fluid and injected over a period of 8–10 s by watching the movement of the meniscus in the calibrated pipette, as previously described (37, 43). After bilateral injections of glutamate, the pipette was cleared and rinsed with distilled water. To inhibit neurons in the NTS, the GABA_A_ receptor agonist muscimol (100 pmol in 100 nL) was nanoinjected into the NTS bilaterally. Inhibition of the NTS neurons that produce sympathoinhibitory reflexes was verified by evoking two reflexes before and after nanoinjections of muscimol into the NTS. Rats received bolus injection of PE (5 µg/kg iv) to activate the arterial baroreflex and phenyl biguanide (PBG) to activate the Bezold-Jarisch reflex (2 µg/kg iv), as previously described (43, 44). At the end of the experiment, the rat was treated with an autonomic ganglionic antagonist (mecamylamine; 5 mg/kg iv) to estimate the minimum SNA. Then, the rat was euthanized with urethane and decapitated.

### Measurement of Plasma Insulin, Cholesterol, and Triglycerides

Blood samples taken from the femoral artery catheter during physiological experiments were collected into heparinized tubes and centrifuged immediately to isolate plasma. Plasma samples were aliquoted and stored at −20°C for analysis by ELISA. Rat Ultrasensitive Insulin ELISA kit (80-INSRTU-E01, ALPCO) was used to quantify plasma insulin concentration. Measurements of plasma triglycerides and total cholesterol concentrations were performed using a Cholesterol E kit (439–17501, Wako Diagnostics) and L-Type TG M reagents (Color A 461–08992, Color B 461–09092 and Multi-Lipid calibrator 464–01601, Wako Diagnostics), respectively. For all plasma measures, the samples were run in duplicate and averaged for each rat. Samples were coded to ensure blinded conditions for performing the assays and analyzing the data.

### Implantation of Glucose Sensing Transmitters

Using aseptic conditions, a laparotomy was performed while the rat was under isoflurane anesthesia. The tip of the transmitter catheter (HD-XG, Data Sciences International, DSI) was inserted rostrally into the abdominal aorta just distal to the level of the kidneys of 11- to 14-wk-old Zucker rats. The aortic wall was sealed around the catheter with a piece of mesh and a small drop of cyanoacrylate adhesive. The catheter and its connected transmitter were secured to the abdominal wall using 4.0-prolene sutures. After the incision was closed, rats were kept warm and monitored until fully conscious. Rats were housed individually, and each cage was placed on a receiver to continuously measure blood glucose by telemetry (DSI), as we have previously reported (38). The transmitters were calibrated using blood samples from a tail snip with rats in fasted and fed states and with glucose tolerance tests. Blood glucose levels were recorded continuously for 2–5 wk.

### Glucose Measures by Telemetry

To calibrate the glucose sensor, a glucose tolerance test was performed 3–4 days after implantation of the glucose transmitter. Rats were fasted for 18 h with access to water and then a baseline blood sample was taken from the tail at ∼8:00 AM. While rats remained in their home cage, the tip of the tail was snipped with sharp scissors to obtain a drop of blood, and the blood was applied to a glucose test strip that was inserted into a calibrated handheld glucometer (Accu-Chek Aviva Plus). One hour later, each rat was briefly lifted from their cage and injected with glucose (1 g/kg from a 0.5 g/mL solution; 0.5–1.0 mL ip). While the rats rested in their home cage, a second blood sample was taken ∼1 min after the peak in glucose measured in real time by telemetry, as recommended by DSI. This method eliminated any underestimation of the change in glucose due to a potential lag time between change in plasma and recorded sensation of glucose by the transmitter. Periodic fed glucose samples were taken from the tail every 1–2 wk to ensure the calibration of the telemetry measures. A second glucose tolerance test was performed after 2–4 wk of telemetry measures to correct for potential drift in measurement of glucose by telemetry. All blood samples from tail snips were performed in duplicate and entered into the telemetry data acquisition software (Ponemah, DSI).

### Data Acquisition and Analysis

Arterial pulse pressure was measured through the femoral artery catheter, and the mean AP and HR were derived from the AP pulse using a low-pass filter (NL110) and a spike trigger (NL201), respectively (Neurolog System, Digitimer). Raw splanchnic SNA was amplified and filtered (10^−3^ kHz with 60 Hz notch filter, differential AC amplifier 1700, A-M Systems). Baseline voltage was obtained by subtraction of voltage due to noise following ganglionic blockade at the end of experiments (37). Raw SNA was full-wave rectified and integrated into 1-s bins, and changes in integrated SNA were measured as percent change from baseline (37). The analog signals were converted to digital form (Micro 1401, Cambridge Electronic Design) to view them online using Spike2 software (Cambridge Electronic Design).

Baroreflex-mediated changes in SNA were fit to a sigmoid curve and analyzed using the Boltzman equation model (Origin 2017; www.originlab.com) (5, 37). In this model: *y* = *A*_2_ + (*A*_1_ − *A*_2_)/{1 + exp [(*x* − *x*_0_/d*x*)]}, where *A*_1_ is the maximum SNA, *A*_2_ is the minimum SNA, *x* is the mean AP, *x*_0_ is the mean AP at the midpoint of the curve (MAP_50_), and d*x* is an estimate of the width of the curve. The maximum gain was calculated as follows: [(*A*_1_ − *A*_2_)/4]/d*x*. The MAP_50_, the gain, and the maximum and minimum SNA were measured for each rat.

Unpaired *t* tests were used to compare baseline parameters, quantified measures for SNA baroreflex curves, total c-Fos+ neurons, and NTS nanoinjections in age-matched OZR and LZR of the same sex. Hourly glucose values, glucose tolerance tests, and counts of c-Fos+ neurons were compared using the appropriate ANOVA followed by Tukey honestly significant difference post hoc tests when a significant *F* value was obtained (SigmaStat, v. 3.5). The significant difference was set at *P* < 0.05. In the tables, group data are displayed as means ± SE. Graphs display individual data points from every subject along with designations for the means and SE.

### Stage of Estrous Cycle in Female Rats

Female rats were not staged by phase of the estrous cycle. The experiments spanned across days and weeks, so the groups were likely to include females in all phases of the 4-day estrous cycle. Because most comparisons were made between female OZRs and LZRs, both groups have estrous cycles. Although the magnitude of the upper plateau and gain of baroreflex-mediated changes in SNA and HR vary across the estrous cycle, the maximum baroreflex-mediated decreases in SNA and HR of the lower plateau do not vary across the estrous cycle (45, 46). This inability to decrease SNA and HR by raising AP is the primary baroreflex-related deficit observed in male OZRs, and maximal baroreflex-mediated inhibition of HR and SNA and activation of the NTS in females was a major focus in the present study.

## RESULTS

### Phenylephrine-Induced Bradycardia and c-Fos Expression in the NTS in Zucker Rats at 12–14 wk of Age

**Figure 1.**
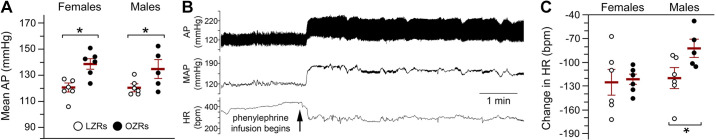
Baseline mean arterial pressure (AP) and baroreflex-mediated bradycardia in conscious 12- to 14-wk-old female and male lean Zucker rats (LZRs) and obese Zucker rats (OZRs). Individual data points are shown with a red horizontal line representing the mean and capped vertical lines representing the SE. *A*: baseline mean AP immediately preceding the infusion of phenylephrine (PE) in female rats (*n *=* *6 LZRs and 6 OZRs) and male rats (*n *=* *6 LZRs and 5 OZRs). *B*: representative recordings of mean AP (MAP), AP, and heart rate (HR) from a female OZR with infusion of PE. *C*: maximum PE-induced decreases in HR in first 5 min of the 40 mmHg rise in AP in rats from *A*. Males and females were analyzed separately by unpaired *t* tests. *P <0.05, OZRs vs. LZRs of same sex.

Body weight was 120% higher in age-matched young adult female OZRs than female LZRs, and body weight was 66% higher in young adult male OZRs than male LZRs (Table 1). The baseline mean AP was significantly higher in conscious OZRs compared with LZRs of the same sex (Fig. 1*A*), but HR was not different in OZRs and LZRs of the same sex (Table 1). Infusion of PE to raise mean AP by ∼40 mmHg evoked an equivalent baroreflex-mediated bradycardia in female OZRs and LZRs (Fig. 1, *B* and *C*). In contrast, baroreflex-mediated bradycardia was significantly smaller in male OZRs compared with male LZRs (Fig. 1*C*), as previously reported (33, 38).

**Table 1.** Baseline values for 12- to 14-wk-old conscious female and male Zucker rats

Group	*n*	Age, days	Body Weight, g	HR, beats/min
Females				
LZRs	6	96.3 ± 2.0	222.7 ± 7.5	369.8 ± 5.9
OZRs	6	96.7 ± 1.9	489.0 ± 16.7*	393.3 ± 10.0
Males				
LZRs	6	90.8 ± 1.3	329.3 ± 15.1	367.2 ± 9.7
OZRs	5	93.2 ± 1.4	545.6 ± 20.8*	353.8 ± 7.5

**Figure 2.**
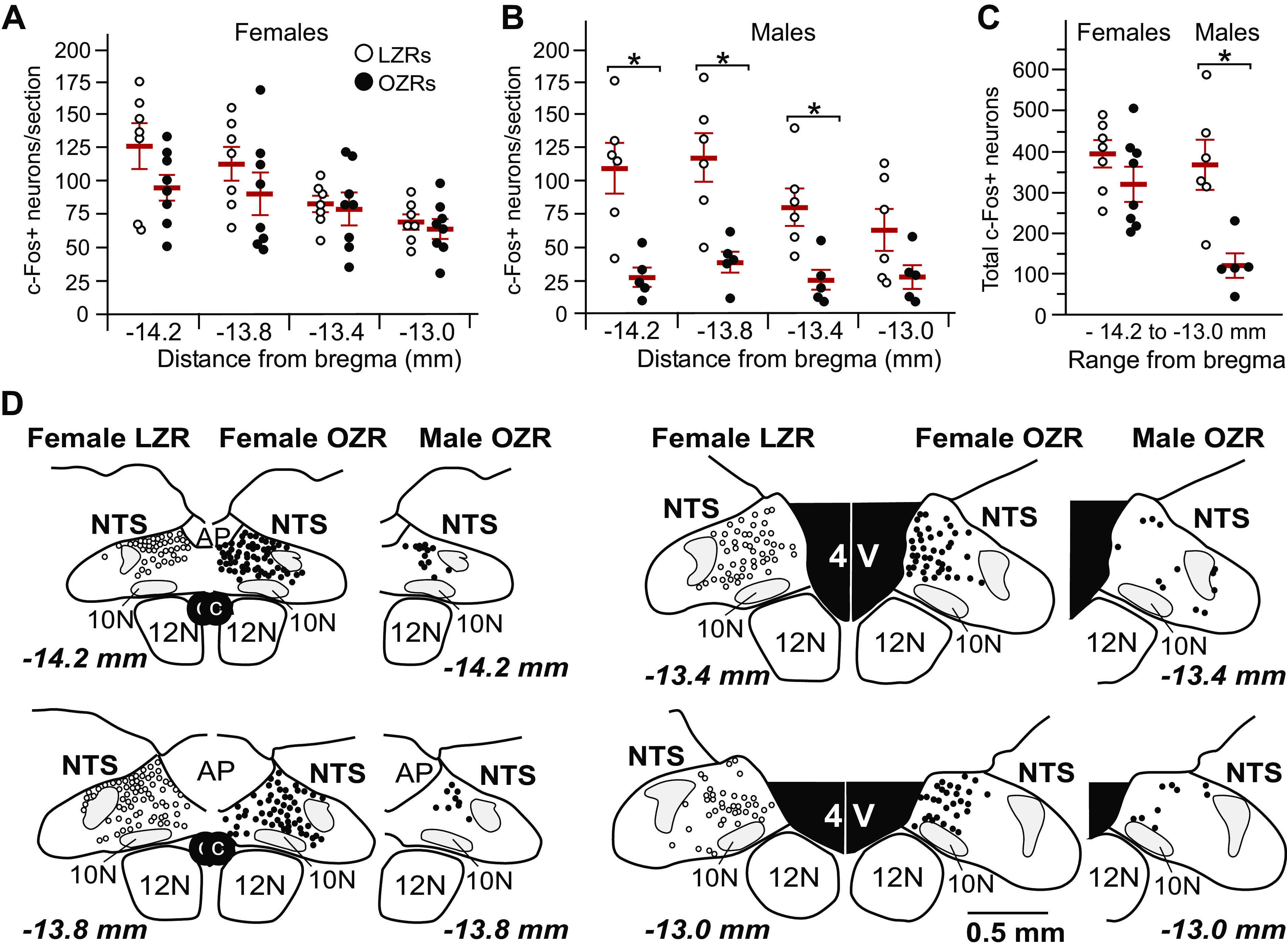
Phenylephrine (PE)-induced c-Fos expression in the nucleus tractus solitarius (NTS) in conscious 12- to 14-wk-old female and male Zucker rats. Individual data points are shown with a red horizontal line representing the mean and capped vertical lines representing the SE. Data are from rats in Fig. 1 and include three additional female rats that were not included in Fig. 1 due to unstable heart rate (HR) readings during the first 5 min of the PE infusion. *A*: counts of c-Fos+ neurons in the NTS of females at four rostro-caudal levels from bregma (*n *=* *8 LZRs and 7 OZRs). *B*: counts of c-Fos+ neurons in the NTS of males at the same four rostro-caudal levels shown in *A* (*n *=* *6 LZRs and 5 OZRs). *C*: total counts of c-Fos+ neurons in the NTS from females and males in *A* and *B*. *D:* representative maps of c-Fos+ neurons in the NTS at four rostro-caudal levels from bregma in a female LZR (*left map inset)*, a female OZR (*middle map inset*), and a male OZR (*right map inset*), as previously reported in male OZRs and LZRs (33). Representative photomicrographs of c-Fos immunohistochemistry from the four groups are in Fig. 3. Males and females were analyzed separately by two-way ANOVA with repeated measures followed by Tukey honestly significant difference (HSD) tests (*A* and *B*) or unpaired *t* tests (*C*). For females there was a main effect for bregma level (*F* = 12.97, *P* < 0.001) in agreement with the expected rostro-caudal distribution of c-Fos+ neurons in the NTS. There was no significant main effect for rat phenotype (*F *=* *1.53, *P* = 0.238) or interaction (*F *=* *1.48, *P* = 0.236), so no pairwise comparisons were made between female OZRs and LZRs. For males, there were significant main effects for bregma level (*F *=* *9.18, *P* < 0.001), phenotype (*F *=* *11.64, *P* = 0.008), and interaction (*F *=* *5.03, *P* = 0.007). *P < 0.05, OZRs vs. LZRs of same sex at same bregma level. LZRs, lean Zucker rats; OZRs, obese Zucker rats.

**Figure 3.**
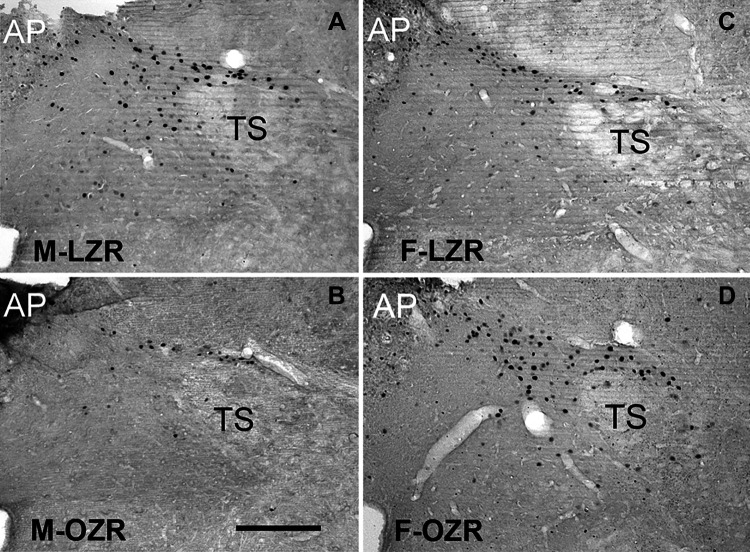
Representative photomicrographs of phenylephrine (PE)-induced c-Fos expression in the nucleus tractus solitarius (NTS) of 12- to 14-wk-old male and female obese Zucker rats (OZRs) and lean Zucker rats (LZRs). The 30-μm sections are in the coronal plane, showing the right half of intermediate NTS at bregma level: 13.8 mm in a male LZR (*A*), a male OZR (*B*), a female LZR (*C*), and a female OZR (*D*), as previously shown in males (33, 38). AP, area postrema; F-LZR, female LZR; F-OZR, female OZR; M-LZR, male LZR; M-OZR, male OZR; TS, tractus solitarius. Scale bar = 250 μm.

Values are means ± SE; *n*, number of rats. Baseline mean arterial pressure (AP) is shown in Fig. 1A. Rats were infused with phenylephrine (PE) to raise mean AP by 40 mmHg to determine maximal baroreflex bradycardia (Fig. 1, *B* and *C*) and c-Fos expression in the nucleus tractus solitarius (NTS) (Figs. 2 and 3). HR, heart rate in beats/min. Males and females were analyzed separately. LZRs, lean Zucker rats; OZRs, obese Zucker rats.

*P < 0.05 vs. LZRs of same sex, unpaired *t* tests.

The 40-mmHg rise in mean AP was maintained for 60–90 min by continued infusion of phenylephrine (PE) to induce c-Fos expression in the NTS. The number of c-Fos+ neurons was comparable in female OZRs and LZRs at each of the four rostro-caudal levels of the NTS examined (Fig. 2*A*) and when the number of cells across the four levels was combined (Fig. 2*C*). In contrast, male OZRs had fewer c-Fos+ neurons at three of four rostro-caudal levels of the NTS examined compared with male LZRs (Fig. 2*B*), yielding an obviously lower total number of c-Fos+ neurons in the NTS in male OZRs (Fig. 2*C*). Representative maps of c-Fos+ neurons in the NTS from a female LZR and a female OZR show similar distributions of c-Fos+ neurons within the four levels of the NTS (Fig. 2*D*). In comparison, the distribution of c-Fos+ neurons in the NTS in a representative male OZR was sparse (Fig. 2*D*). Distribution of c-Fos+ neurons in the NTS of male LZRs was similar to our previous study (33) and resembled the distributions observed in the female Zucker rats. Figure 3 shows representative photomicrographs of c-Fos expression in the NTS at 13.8 mm caudal to bregma from male and female OZRs and LZRs.

### Baroreflex-Induced Changes in SNA in Female Rats at 15–16 wk of Age

**Figure 4.**
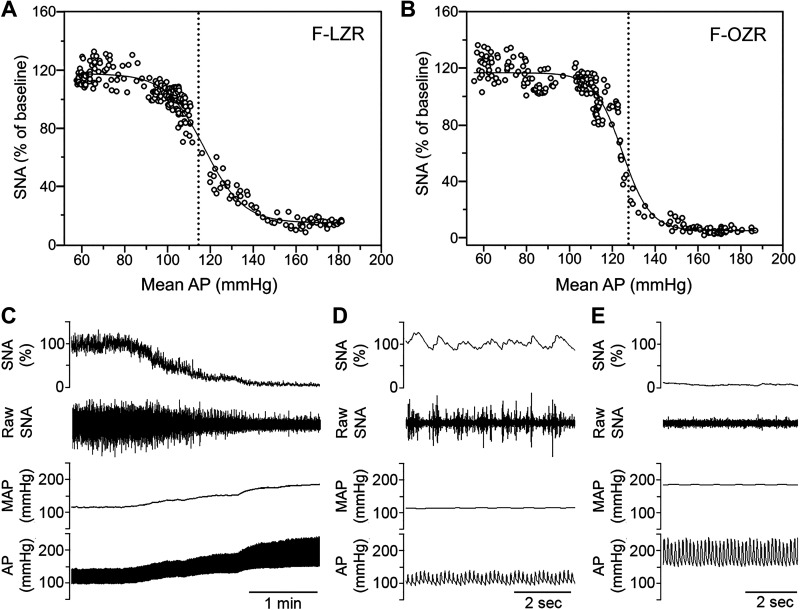
Representative sympathetic baroreflexes from urethane-anesthetized, 15-wk-old female rats. Changes in splanchnic sympathetic nerve activity (SNA) were produced by raising mean arterial pressure (AP) with phenylephrine (PE) and decreasing mean AP with sodium nitroprusside and analyzed using the Boltzman equation. *A:* sigmoid curve from one female lean Zucker rat (F-LZR) with 253 points of data. *B:* sigmoid curve from one female obese Zucker rat (F-OZR) with 237 points of data. The vertical dotted line denotes the mean AP at the midpoint of the curve (MAP_50_). *C*: representative tracings from a female OZR showing a PE-induced rise in AP and baroreflex-mediated inhibition of SNA. Expanded time scale from rat (*C*) shows baseline SNA (*D*), and the PE-induced nadir of SNA (*E*). Group baseline data are in Table 2, and group baroreflex data are in Table 3.

In age-matched young adult female LZRs and OZRs, body weight was higher in OZRs compared with LZRs (Table 2). As previously reported in male Zucker rats (37), baseline splanchnic SNA and mean AP were higher in urethane-anesthetized female OZRs compared with LZRs (Table 2). Infusion of PE raised AP and produced a nadir of SNA that occurred in ∼2 min (Fig. 4, *C–E*; 126.0 ± 19.7 s in LZRs and 104.6 ± 18.6 s in OZRs). Infusion of sodium nitroprusside lowered AP and produced a peak in SNA that occurred within 1 min (54.6 ± 11.7 s in LZRs and 60.0 ± 6.11 s in OZRs). The changes in SNA over the full range of mean AP were fit to a sigmoid curve for analysis, as shown in a representative female LZR and OZR (Fig. 4, *A* and *B*, Table 3). As previously observed in male OZRs (5), the higher baseline mean AP coincided with a rightward shift in the mean AP at the midpoint of the curve (MAP_50_) in female OZRs compared with LZRs (Table 3). In contrast, unlike the impaired sympathetic baroreflex efficacy in male OZRs (5), the baroreflex gain and range of SNA were not different in female LZRs and OZRs (Table 3). In agreement with the equivalent PE-induced bradycardia in conscious female OZRs and LZRs (Fig. 1*C*), maximal baroreflex-mediated inhibition of SNA was not different in anesthetized female OZRs and LZRs (Fig. 4 and Table 3). These data suggest that in young adult female OZRs, the higher baseline mean AP and adaptive rightward shift in MAP_50_ occur in the absence of diminished baroreflex-mediated changes in SNA and HR.

**Table 2.** Baseline values for 14- to 16-wk-old anesthetized female and male Zucker rats

Group	*n*	Age, days	Body Weight, g	SNA, μV	Mean AP, mmHg	HR, beats/min
Females						
LZRs	5	108.6 ± 0.9	230.4 ± 4.2	0.98 ± 0.10	94.1 ± 4.7	436.6 ± 7.3
OZRs	5	107.8 ± 0.6	466.0 ± 5.0*	2.25 ± 0.40*	113.4 ± 2.9*	412.5 ± 14.4
Males						
LZRs	6	106.0 ± 2.2	381.8 ± 13.3	1.37 ± 0.09	107.0 ± 4.8	385.6 ± 17.6
OZRs	6	106.0 ± 2.0	580.7 ± 41.5*	2.17 ± 0.16*	121.4 ± 4.2*	378.4 ± 12.6

**Figure 5.**
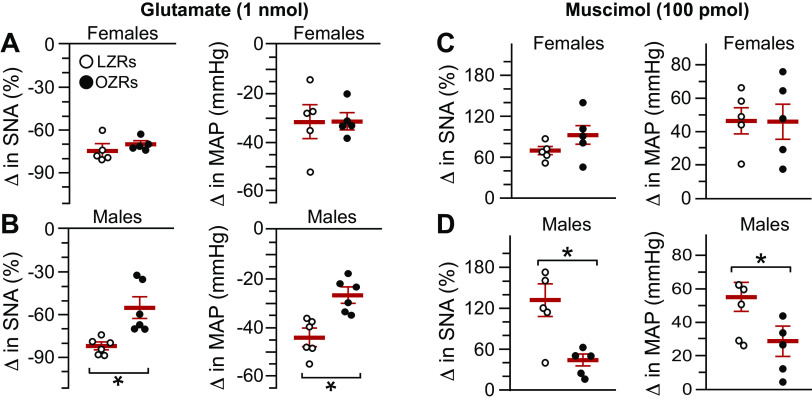
Physiological responses to activation and inhibition of neurons in the nucleus tractus solitarius (NTS) in urethane-anesthetized female and male obese Zucker rats (OZRs) and lean Zucker rats (LZRs). Individual data points are shown with a red horizontal line representing the mean and capped vertical lines representing the SE*. A*: nanoinjections of glutamate (1 nmol/50 nL/side with responses averaged) evoked comparable decreases in sympathetic nerve activity (SNA) and mean arterial pressure (MAP) in female OZRs and LZRs (*n *=* *5/group). *B*: glutamate evoked smaller decreases in SNA and MAP in male OZRs compared with male LZRs (*n* = 6/group). *C*: bilateral nanoinjections of GABA_A_ agonist muscimol (100 pmol/100 nL/side) into the NTS produced comparable decreases in SNA in the same female OZRs and LZRs. *D*: muscimol produced smaller rises in SNA and MAP in the same male OZRs compared with male LZRs. Males and females were analyzed separately by unpaired *t* tests for each measure. *P < 0.05, OZRs vs. LZRs of same sex. Representative recordings of physiological responses to muscimol in the NTS are in Fig. 6.

**Figure 6.**
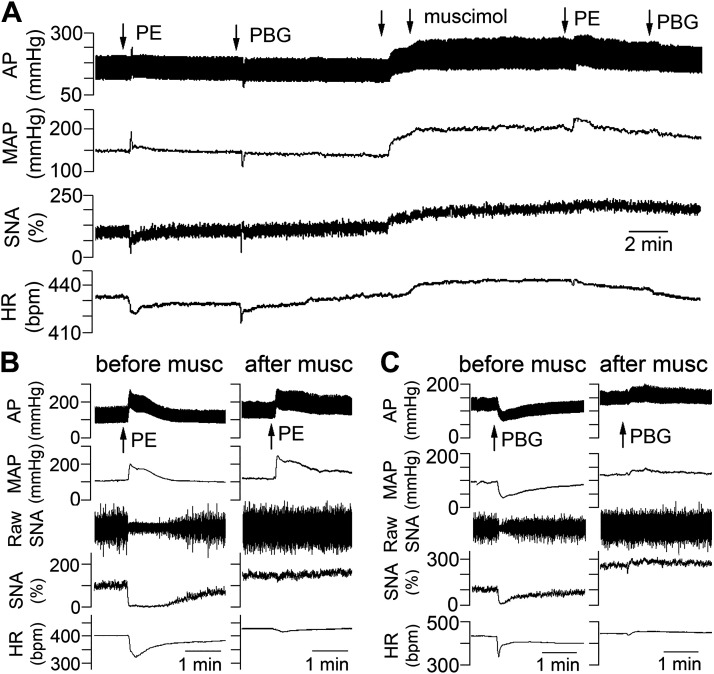
Representative recordings of physiological responses to inhibition of neurons in the nucleus tractus solitarius (NTS) produced by bilateral nanoinjections of muscimol. *A:* nanoinjections of muscimol (2 downward arrows, left then right side) evoked rises in arterial pressure (AP), sympathetic nerve activity (SNA), and heart rate (HR) in a urethane-anesthetized male lean Zucker rat (LZR). To verify effective inhibition of the NTS, intravenous injections of phenylephrine (PE) (5 µg/kg in 50 µL, at downward arrow) and phenyl biguanide (PBG, 2 µg/kg in 50 µL, at downward arrow) were performed before and after nanoinjections of muscimol into the NTS. *B:* expanded time scale to show PE-induced rise in AP and baroreflex-mediated decrease in SNA and HR before muscimol and the absence of PE-induced baroreflex-mediated changes in SNA and HR after muscimol. *C*: expanded time scale to show PBG-induced decreases in AP, SNA, and HR before muscimol and the absence of PBG-induced changes in AP, SNA, and HR after muscimol. Group data for muscimol-induced changes in AP, SNA, and HR are in Fig. 5.

Values are mean ± SE; *n*, number of rats. Rats were anesthetized with urethane, artificially ventilated, and paralyzed. Sympathetic baroreflexes in females are shown in Fig. 4 and Table 3. Physiological responses to nanoinjections of glutamate and muscimol in males and females are shown in Figs. 5 and 6. AP, arterial pressure; HR, heart rate; LZRs, lean Zucker rats; OZRs, obese Zucker rats; SNA, sympathetic nerve activity;. Males and females were analyzed separately. *P < 0.05 vs. LZRs of same sex, unpaired *t* tests.

**Table 3.** Sympathetic baroreflexes in 15- to 16-wk-old anesthetized female Zucker rats

Group	*n*	MAP_50_, mmHg	Slope, %/mmHg	Lower Plateau, % SNA	Upper Plateau, % SNA	Range, % SNA
LZRs	5	117.3 ± 5.2	−2.8 ± 0.4	14.0 ± 4.3	123.5 ± 9.9	109.5 ± 9.2
OZRs	5	130.5 ± 1.9*	−2.2 ± 0.6	13.9 ± 3.7	131.4 ± 11.4	117.5 ± 11.5

Values are means ± SE; *n*, number of rats. Rats were anesthetized with urethane, artificially ventilated, and paralyzed. Baseline values for these rats are in Table 2. Changes in mean arterial pressure (AP) and sympathetic nerve activity (SNA) were measured during infusions of phenylephrine (PE) and nitroprusside. The MAP50 is the mean AP at the midpoint of the sigmoid curve. Representative examples of the relationship between mean AP and SNA in one female lean Zucker rat (LZR) and obese Zucker rat (OZR) are in Fig. 4. *P < 0.05 vs. LZRs, unpaired *t* tests.

### Nanoinjections into the NTS of Zucker Rats at 14–16 wk of Age

Neurons in the NTS were activated by nanoinjection of glutamate on each side (1 nmol in 50 nL), and the maximum changes in SNA and mean AP from both sides were averaged for each rat. Nanoinjection of glutamate into NTS evoked decreases in SNA and mean AP that were not different in 15- to 16-wk-old female LZRs and OZRs (Fig. 5*A*). In contrast, smaller glutamate-induced decreases in SNA and mean AP were observed in 14- to 16-wk-old male OZRs compared with male LZRs (Fig. 5*B*), as previously reported (33). Inhibition of neurons in the NTS with bilateral nanoinjections of the GABA_A_ agonist muscimol produced comparable rises in SNA and mean AP in female LZRs and OZRs (Fig. 5*C*). In contrast, nanoinjections of muscimol into the NTS produced smaller rises in SNA and mean AP in male OZRs compared with male LZRs (Fig. 5*D* and Fig. 6*A*), suggesting reduced tonic inhibition of SNA and AP by the NTS coincident with impaired activation. The efficacy of the inhibition of the NTS was verified by the absence of changes in SNA and HR after injections of PE (5 µg/kg iv) and PBG (2 µg/kg iv), as previously reported (43, 44). Before inhibition of the NTS, the PE-induced rise in AP produced prominent baroreflex-mediated decreases in SNA and HR that were absent after inhibition of the NTS (Fig. 6, *A* and *B*). Likewise, before inhibition of the NTS, the injection of PBG evoked significant decreases in SNA, HR, and AP that were abolished by inhibition of the NTS (Fig. 6, *A* and *C*).

### Metabolic Attributes in Plasma of Zucker Rats at 14–16 wk of Age

Compared with young adult male LZRs, age-matched male OZRs had exaggerated weight gain (45.4% higher) and elevated plasma insulin, total cholesterol, and triglycerides (Table 4), as expected (38). With access to food, morning blood glucose levels were 85% higher in male OZRs compared with male LZRs (Table 4). Young adult female OZRs also had exaggerated weight gain (114.9% higher) in comparison to age-matched female LZRs (Table 4). As seen in male OZRs, female OZRs had significantly elevated plasma insulin, total cholesterol, and triglycerides compared with age-matched female LZRs (Table 4). In contrast, with access to food, morning blood glucose not different between female LZRs and OZRs (Table 4).

**Table 4.** Metabolic attributes of 14- to 16-wk-old male and female Zucker rats

Group	Age, days	Body Weight, g	Insulin, ng/dL	Total Cholesterol, mg/dL	Triglycerides, mg/dL	Fed Glucose, mg/dL
Males						
LZRs	110.2 ± 2.8	395.2 ± 13.0	1.5 ± 0.4	77.0 ± 6.6	54.5 ± 12.3	117.8 ± 2.1
	(6)	(6)	(6)	(6)	(6)	(6)
OZRs	108.3 ± 1.2	574.8 ± 20.4*	7.1 ± 1.6*	181.2 ± 16.7*	422.1 ± 76.7*	217.9 ± 34.4*
	(12)	(12)	(5)	(5)	(5)	(7)
Females						
LZRs	109.7 ± 1.2	226.4 ± 2.5	1.0 ± 0.1	69.8 ± 4.6	102.3 ± 18.9	108.2 ± 1.6
	(14)	(14)	(8)	(8)	(6)	(9)
OZRs	108.8 ± 1.2	486.5 ± 8.7*	7.4 ± 2.0*	119.8 ± 6.8*	567.4 ± 85.4*	113.6 ± 3.5
	(15)	(15)	(7)	(7)	(5)	(11)

Values are means ± SE. Group sizes are shown in parentheses below the data. Plasma measures for insulin, total cholesterol, and triglycerides were performed using blood drawn from a femoral artery catheter in isoflurane-anesthetized rats. Because anesthesia significantly alters blood glucose values, blood samples for fed glucose were taken from a tail snip while the rat was in their home cage before undergoing anesthesia. All samples were taken in the morning (9:00–11:00 AM). Males and females were analyzed separately. LZRs, lean Zucker rats; OZRs, obese Zucker rats.

*P < 0.05 vs. LZRs of same sex and age, unpaired *t* tests.

### Blood Glucose Measured by Telemetry in Zucker Rats at 12–14 wk and 15–17 wk of Age

**Figure 7.**
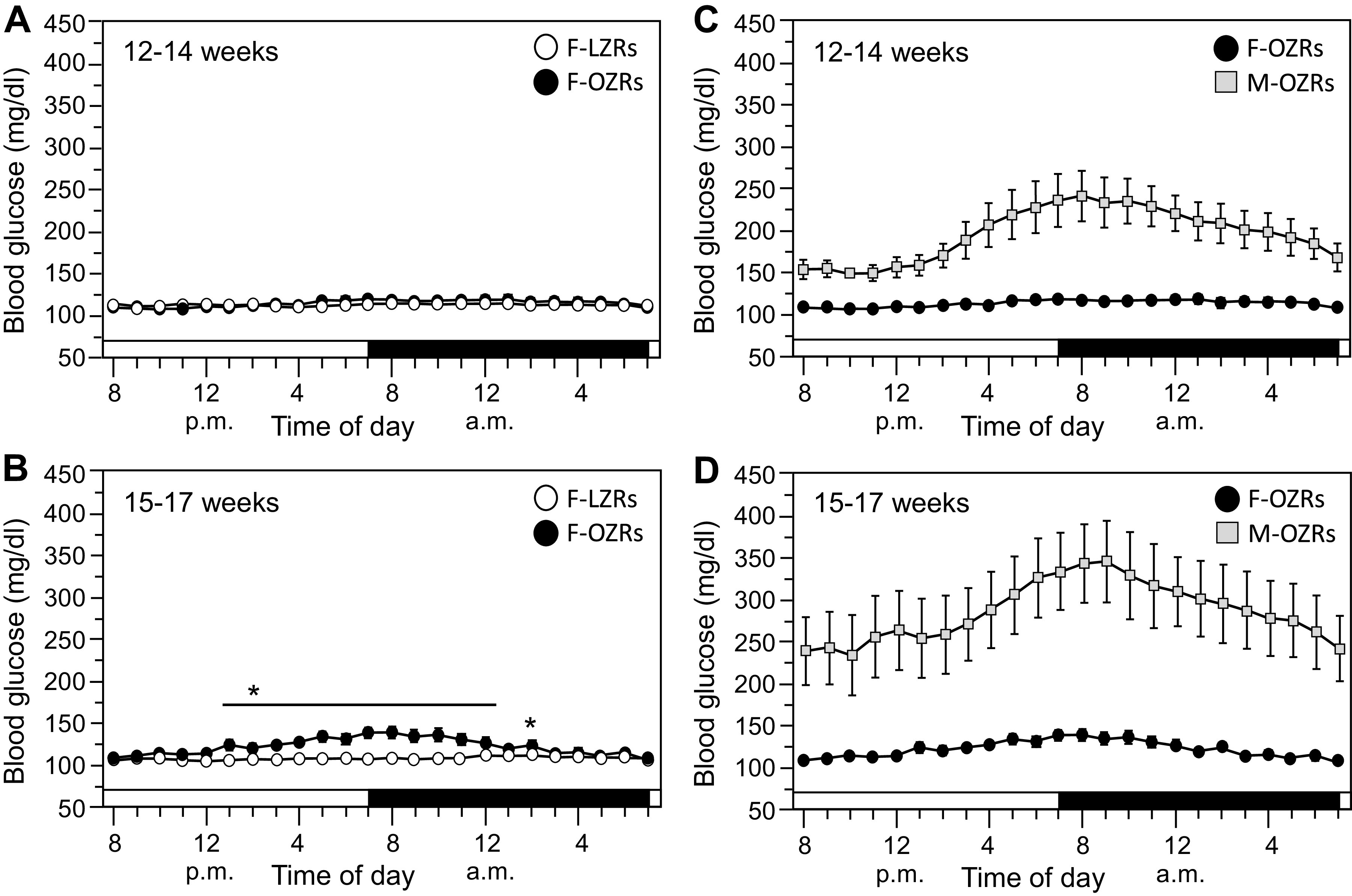
Hourly average blood glucose measurements by telemetry in conscious rats with access to food at two age ranges. Data are means ± SE. Left panels compare female lean Zucker rats (F-LZRs) and female obese Zucker rats (F-OZRs), and right panels compare the same female OZRs with male OZRs (M-OZRs). *A*: hourly blood glucose over a 24-h period in 12- to 14-wk-old F-OZRs (*n *=* *13) and F-LZRs (*n *=* *13). *B*: hourly blood glucose over a 24-h period in 15- to 17-wk-old F-LZRs and F-OZRs from *A* without one of the OZRs (*n *=* *12). *C*: hourly blood glucose over a 24-h period in 12- to 14-wk-old F-OZRs from *A* (*n *=* *13) and M-OZRs (*n *=* *12). *D:* hourly blood glucose over a 24-h period in 15- to 17-wk-old F-OZRs from *B* (*n *=* *12) and in six of the male OZRs from *C*. Hourly values are the mean of twelve 5-min samples occurring 30 min preceding the hour to 30 min after the hour. Data were analyzed for each age range using a two-way ANOVA with repeated measures for time and Tukey’s honestly significant difference (HSD) tests when significant *F* values were observed. The second factors for rat phenotype (in *A* and *B*) or sex (in *C* and *D*) were analyzed separately within age range. *P < 0.05, age-matched F-OZRs vs. F-LZRs at that time point in *B.* In *C* and *D*, blood glucose was significantly higher at all time points in M-OZRs vs. F-OZRs within age range. The white and black bars across the *x*-axis denote periods of lights on and lights off, respectively.

Hourly averages of blood glucose were measured by telemetry over a 24-h period while rats had access to food and water. In 12- to 14-wk-old female OZRs and LZRs, blood glucose was equivalent at every hour over the 24-h period (Fig. 7*A*). By 15–17 wk of age, female OZRs showed modest elevations in hourly averages of blood glucose for 12 h of the 24-h period (Fig. 7*B*). To place the modest, periodic rises in blood glucose observed in 15- to 17-wk-old female OZRs into perspective, these female OZRs were compared with age-matched male OZRs. At 12–14 wk of age, male OZRs had markedly elevated blood glucose, which was much higher at every hour of the day and night compared with female OZRs (Fig. 7*C*). By 15–17 wk of age, the modest elevation of blood glucose observed during some hours of the day in female OZRs was minimal compared with the prominently elevated blood glucose observed at all hours of the day and night in age-matched male OZRs (Fig. 7*D*).

### Glucose Tolerance Tests in Female Zucker Rats at 12–17 wk of Age

After 18 h of overnight fasting with access to water, rats were injected with glucose (1 g/kg ip) to determine their glucose tolerance. To best demonstrate the emerging development of glucose intolerance over this age range, the rats were separated into three age ranges, namely 12–13, 14–15, and 16–17 wk of age. Within each age range, female OZRs weighed more than female LZRs and both phenotypes showed an increase in body weight with age (Table 5). Fasting blood glucose was comparable in female OZRs and LZRs at 12–13 and 16–17 wk of age (Table 5). Although fasting glucose was slightly but significantly higher in female OZRs compared with LZRs at 14–15 wk of age, across the three age ranges, there were no significant differences in fasting blood glucose within rat phenotype (Table 5).

**Table 5.** Glucose tolerance tests in 12- to 17-wk-old female Zucker rats

Group	*n*	Age, days	Body Weight, g	Fasting Glucose, mg/dL	Peak Glucose, mg/dL	Area Under the Curve, mg/dL × 3 h
12- to 13-wk old:						
LZRs	5	94.3 ± 0.2	204.7 ± 5.3	92.1 ± 2.0	235.0 ± 13.7	201.9 ± 9.6
OZRs	6	94.3 ± 0.2	426.5 ± 14.6*	99.1 ± 6.0	248.8 ± 24.0	283.1 ± 42.5
14- to 15-wk old						
LZRs	9	106.0 ± 1.1	210.8 ± 3.7	87.3 ± 2.5	234.9 ± 11.4	273.8 ± 36.0
OZRs	9	105.1 ± 1.0	457.0 ± 15.6*	100.0 ± 3.4*	302.2 ± 12.5*	419.1 ± 38.6*
16- to 17-wk old						
LZRs	8	116.0 ± 1.6	243.6 ± 6.5†	92.8 ± 2.3	246.6 ± 21.4	219.2 ± 20.6
OZRs	10	117.1 ± 1.2	490.8 ± 9.4*#	96.0 ± 2.9	347.4 ± 18.2*†	477.1 ± 53.0*†
*P* values						
Rat phenotype			<0.001	0.008	<0.001	<0.001
Age range			<0.001	0.877	0.014	0.033
Interaction			0.451	0.339	0.068	0.114

**Figure 8.**
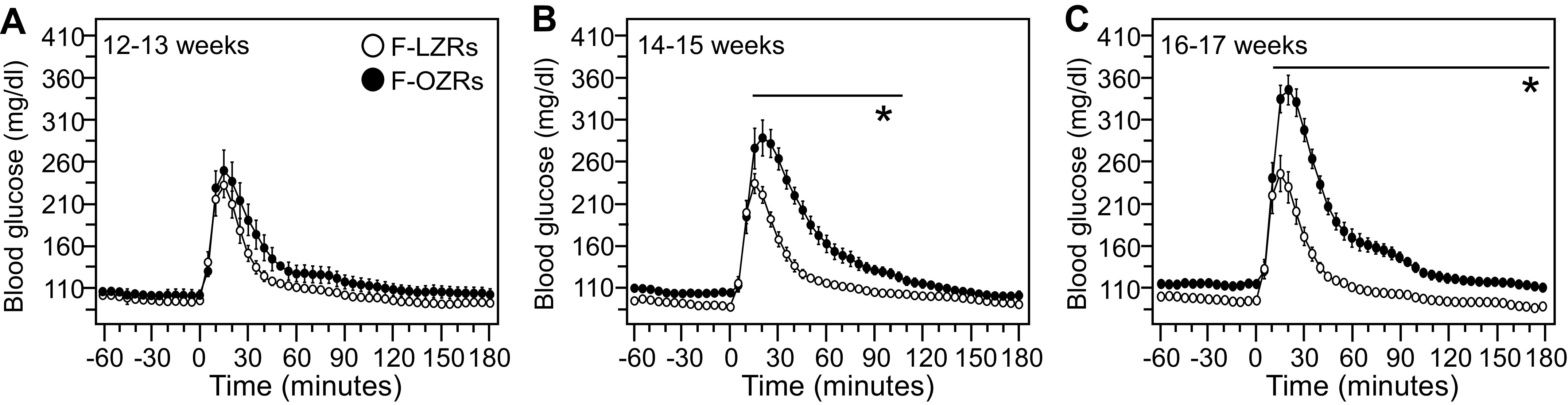
Glucose tolerance tests in female obese Zucker rats (OZRs) and lean Zucker rats (LZRs) with blood glucose measured by telemetry at three age ranges. Data are presented as means ± SE. The age ranges of rats were 12–13 wk (*A*), 14–15 wk (*B*), and 16–17 wk (*C*). *A*–*C*: blood glucose values 60 min before and 180 min after injection of glucose (*time 0*) measured by telemetry in 5-min increments at three age ranges. Each age range was analyzed by two-way ANOVA with repeated measures followed by Tukey honestly significant difference (HSD) tests when a significant *F* value was obtained for rat phenotype. There was no significant effect for phenotype in (*A*) *F *=* *2.27, *P* = 0.16. There was a significant effect for phenotype in (*B*) *F *=* *29.82, *P* < 0.001 and (*C*) *F *=* *37.72, *P* < 0.001. *P < 0.05, F-OZRs vs. F-LZRs at those time points. All groups had a significant main effect for time. See Table 5 for group sizes and analyses of single value comparisons during the glucose tolerance tests at each age range.

Values are means ± SE; *n* = group size. Time courses for the glucose tolerance tests in these rats are shown in Fig. 8. Fasting glucose values were derived from telemetry measures from 145 to 60 min before the glucose tolerance test, immediately before tail samples were taken to calibrate the telemetry sensors. Peak glucose values are the highest 5-min glucose value measured by telemetry in each rat after injection of glucose. Area under the curve was derived from time 0 after the injection of glucose to 180 min after injection of glucose. Data were analyzed by two-way ANOVAs with Turkey HSD post hoc tests when a significant F value was achieved for phenotype or age. LZRs, lean Zucker rats; OZRs, obese Zucker rats.

*P < 0.05 vs. LZRs in same age range. †*P* < 0.05 vs. 12- to 13-wk-old rats of same phenotype. #*P* < 0.05 vs. OZRs in both of the other age ranges.

In female LZRs, the peak in blood glucose and the area under the curve did not differ across the three age ranges (Table 5). In contrast, female OZRs at 16–17 wk had a higher peak in blood glucose and a larger area under the curve than in younger female OZRs (Table 5). Comparisons of glucose tolerance measures between age-matched female LZRs and OZRs showed an emergence of mild glucose intolerance in female OZRs. At 12–13 wk of age, all glucose tolerance measures were equivalent in female LZRs and OZRs (Fig. 8*A* and Table 5), in contrast to the pronounced glucose intolerance in male OZRs at 12–14 wk of age (38). By 14–15 wk of age, female OZRs had a higher peak in blood glucose, a larger area under the curve, and a longer recovery time compared with female LZRs (Fig. 8*B* and Table 5). These differences became more prominent in 16- to 17-wk-old female LZRs and OZRs (Fig. 8*C* and Table 5). Blood glucose levels recovered to levels seen in age-matched female LZRs in 105 min in 14- to 15-wk-old female OZRs (Fig. 8*B*) and in 180 min in 16- to 17-wk-old female OZRs (Fig. 8*C*). Although no direct comparisons were made for glucose tolerance test values between male and female OZRs in the present study, we previously reported much higher values in male OZRs at 15–17 wk of age (e.g., The AUC was 940.6 ± 138.5 vs. 310.08 ± 80.6 mg/dL × 3 h in male OZRs and LZRs, respectively) (38).

### Metabolic Attributes in Plasma of Female Zucker Rats at 24–27 wk of Age

The difference in body weights between female OZRs and LZRs continued to rise with age. At 24–27 wk of age, female OZRs weighed 148% more than age-matched LZRs, whereas 14- to 16-wk-old female OZRs weighed 115% more than age-matched LZRs (Tables 4 and 6). As seen in rats at 14–16 wk of age, at 24–27 wk of age, female OZRs had markedly elevated plasma insulin, total cholesterol, and triglycerides compared with age-matched female LZRs (Table 6). Fed glucose levels were modestly elevated in female OZRs compared with age-matched female LZRs, but similar to morning blood glucose values in 12- to 14-wk-old female OZRs and LZRs (Fig. 7 and Table 4 vs. 6).

**Table 6.** Metabolic attributes of 24- to 27-wk-old female LZRs and OZRs

Group	Age, days	Body Weight, g	Insulin, ng/dL	Total Cholesterol, mg/dL	Triglycerides, mg/dL	Fed Glucose, mg/dL
F-LZRs	185.5 ± 1.7	276.0 ± 7.1	0.8 ± 0.1	88.0 ± 5.4	138.5 ± 21.5	108.4 ± 1.9
	(12)	(12)	(12)	(12)	(12)	(10)
F-OZRs	184.5 ± 2.0	682.7 ± 13.7*	7.3 ± 1.7*	480.5 ± 60.4*	843.8 ± 45.4*	124.2 ± 5.2*
	(10)	(10)	(10)	(10)	(10)	(6)

Values are means ± SE. Group sizes are in parentheses below the data. For measures of plasma insulin, total cholesterol, and triglycerides, blood samples were taken from conscious rats through a femoral artery catheter before infusion of phenylephrine (PE). As performed in younger rats (Table 4), blood samples for fed glucose were taken from a tail snip while the rat was in their home cage. F-LZRs, female lean Zucker rats; F-OZRs, female obese Zucker rats.

*P < 0.05 vs. LZRs, unpaired *t* tests.

### PE-Induced Bradycardia and c-Fos Expression in the NTS in Female Zucker Rats at 24–27 wk of Age

**Figure 9.**
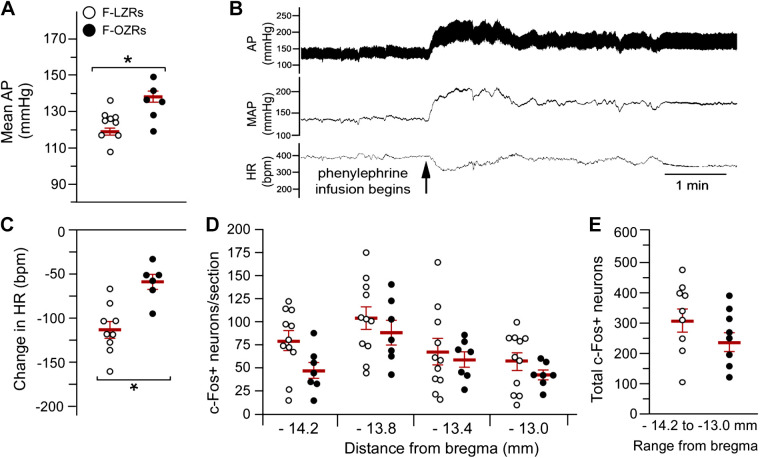
Baseline mean arterial pressure (AP) and baroreflex-mediated bradycardia in conscious 24- to 27-wk-old female lean Zucker rats (F-LZRs) (*n* = 9) and obese Zucker rats (F-OZRs) (*n* = 6). Values are means ± SE. *A*: baseline mean AP immediately preceding the infusion of phenylephrine (PE). *B*: representative recordings of AP, mean AP (MAP), and heart rate (HR) from a female OZR with infusion of PE. *C*: maximum PE-induced decreases in HR in the first 5 min of the rise in AP in rats from *A*. *D*: counts of c-Fos+ neurons in the nucleus tractus solitarius (NTS) at four rostro-caudal levels from bregma. This graph contains three additional rats (*n *=* *11 F-LZRs and 7 F-OZRs) that are not included in (*A*) due to unstable HR at the beginning of the infusion of PE. *E*: total counts of c-Fos+ neurons from the four rostro-caudal levels in *D*. Data in *A*, *C*, and *E* were analyzed by unpaired *t* tests. *P <0.05, F-OZRs vs. F-LZRs. Data in *D* were analyzed by two-way ANOVA with repeated measures for bregma level. There was a significant main effect for bregma level (*F *=* *9.645, *P* < 0.001), consistent with the expected distribution of barosensitive NTS neurons. There was no significant main effect for phenotype (*F *=* *1.461, *P* = 0.248) or interaction (*F *=* *0.428, *P* = 0.734), so no pair-wise comparisons were made for F-OZRs and F-LZRs in *D.*

In aged-matched female LZRs and OZRs (186.1 ± 1.7 and 184.8 ± 2.5 days), the OZRs weighed 134% more than the LZRs (654.3 ± 21.3 vs. 279.2 ± 7.1, *P < 0.05, unpaired *t* test). As seen in females at 12–14 wk of age, the baseline mean AP was higher in female OZRs compared with LZRs at 24–27 wk of age (Fig. 9*A*). In contrast to the younger females, at 24–27 wk of age, infusion of PE to raise mean AP by ∼40 mmHg evoked a much smaller decrease in HR in female OZRs compared with female LZRs (Fig. 9, *B* and *C*). Although the impaired baroreflex efficacy in the older female OZRs resembled the deficit seen in young adult male OZRs (Fig. 1*C*), a PE-induced rise in mean AP did not yield the same outcome for activation of the NTS. Whereas 12- to 14-wk-old male OZRs had significantly fewer PE-induced c-Fos+ neurons than age-matched LZRs (Fig. 2, *B* and *C*), PE infusion to raise mean AP by ∼40 mmHg in 24- to 27-wk-old female OZRs and LZRs produced comparable numbers of c-Fos+ neurons at each of the four rostro-caudal levels of the NTS examined (Fig. 9*D*), yielding equivalent total number of c-Fos+ neurons in the NTS of 24- to 27-wk-old female OZRs and LZRs (Fig. 9*E*).

## DISCUSSION

Metabolic syndrome comprises a cluster of adverse cardiovascular and metabolic traits associated with excess adiposity. Compared with women, a similar degree of obesity in men is associated with more severe dyslipidemia, hyperinsulinemia, impairment of glycemic control, and elevated SNA and AP (19, 21–23). As seen in men with obesity, 3- to 4-mo-old male OZRs develop sympathetically driven hypertension and impaired baroreflexes with insulin resistance and hyperglycemia (4, 5, 11, 12, 19, 22, 23, 38, 43). In these male OZRs, treatments that abolish hyperglycemia improve impaired baroreflex-mediated activation of the NTS and bradycardia despite persistent hypertension (38). In contrast, hypertensive female OZRs have normal baroreflex-mediated bradycardia at 3 mo of age that is later diminished by 6 mo of age (36). The major new findings of this study are that PE-induced c-Fos expression in the NTS and sympathetic baroreflex efficacy were also equivalent in 3- to 4-mo-old female OZRs and LZRs, despite elevated SNA and AP in female OZRs. Activation and inhibition of neurons in the NTS also evoked comparable changes in SNA and AP in female OZRs and LZRs, whereas these responses were reduced in male OZRs. At 3–4 mo of age, male and female OZRs had excess weight gain with elevated plasma insulin and lipids, but only male OZRs had pronounced hyperglycemia with access to food. At 6 mo of age, the development of impaired baroreflex-mediated bradycardia in female OZRs was not accompanied by pronounced hyperglycemia or diminished PE-induced c-Fos expression in the NTS. These data suggest that better glycemic control in young adult female OZRs protects baroreflex-mediated activation of the NTS and baroreflex efficacy. In contrast, later development of diminished baroreflex-mediated bradycardia in female OZRs occurs by mechanisms unrelated to elevated blood glucose or diminished activation of the NTS.

We quantified NTS neurons that expressed c-Fos protein after a PE-induced rise in AP to provide an index of recruitment and further excitation of tonically active barosensitive NTS neurons (14, 40, 47). Limitations of using c-Fos expression include an inability to measure how the rise in AP affects individual NTS neurons or cellular mechanisms for observed differences in c-Fos expression across groups. An advantage of the c-Fos measure is the ability to observe a population response throughout the NTS in conscious rats. Baroreceptor afferent nerves to the NTS are the primary stimulus for PE-induced c-Fos expression in the NTS, because denervation of these nerves from the aortic arch and carotid sinuses virtually eliminates PE-induced c-Fos+ NTS neurons (47). Most of the PE-induced c-Fos expression occurs in glutamatergic NTS neurons located in regions that receive baroreceptor afferent inputs (40). These glutamatergic c-Fos+ neurons (∼30%) project to the caudal ventrolateral medulla, suggesting a portion of the activated neurons is part of the brain stem baroreflex pathway (40, 48). In contrast, very few PE-induced c-Fos+ neurons in the NTS project to the rostral ventrolateral medulla, suggesting the activated neurons are not part of the peripheral chemoreflex pathway (40). In the present study, a large rise in AP was used to maximally reduce SNA or HR, because the lower plateau of the baroreflex is clearly impaired in young adult male OZRs but not in female OZRs (5, 33, 36, 37). Phenylephrine-induced c-Fos expression in NTS neurons, NTS-mediated reductions in SNA and AP, and baroreflex efficacy are comparable in male OZRs and LZRs, and all of these responses become diminished in young adult male OZRs (5, 33). Treatments that increase PE-induced c-Fos expression in the NTS also improve baroreflex efficacy in male OZRs (38). Not surprisingly, in the present study, PE infusion produced an equivalent number of c-Fos+ NTS neurons in young adult female OZRs and LZRs coincident with an absence of differences in baroreflex efficacy. Unexpectedly, older adult female OZRs had impaired baroreflex bradycardia in the absence of diminished c-Fos expression in the NTS.

Hypertension and impaired baroreflex efficacy are strongly correlated and both are worsened by obesity (11). However, normotensive people with obesity can also have diminished baroreflex-mediated control of HR and SNA, suggesting additional mechanisms for obesity-related impairment of baroreflexes (11, 12). Adult male and female OZRs develop elevated SNA and AP and impaired baroreflex efficacy, but the relative age of onset differs by sex (5, 36). Like male rats made obese by a high-fat diet, male OZRs develop diminished baroreflex efficacy before the onset of hypertension (5, 49). In contrast, female OZRs develop diminished baroreflex-mediated bradycardia long after the onset of hypertension (36). In both cases, the relative onset of impaired baroreflex efficacy and hypertension occur independently, strongly suggesting that these two traits occur by distinct mechanisms, as seen in some humans with obesity (11, 12). In young adult Zucker rats, although diminished baroreflex efficacy was only present in male OZRs, male and female OZRs both had a rightward shift in the MAP_50_ in their sympathetic baroreflex curve coincident with a higher baseline AP (5). Intriguingly, this adaptive baroreflex resetting is not apparent in baroreceptor afferent nerve activity of young adult male OZRs. The threshold AP for the onset of aortic depressor nerve activity and changes in this baroreceptor afferent nerve activity in relation to AP are equivalent in young adult male OZRs and LZRs, whether the nerve activity is measured as rectified voltage or percent change from baseline (37). These data suggest that the baroreflex resetting occurs by mechanisms beyond the afferent nerves, as seen with many forms of hypertension (14). In agreement with an absence of baroreceptor afferent nerve resetting, baseline aortic depressor nerve activity and AP are both higher in male OZRs compared with male LZRs (37). In male and female OZRs, the adaptive shift of the baroreflex curve to a higher AP appears to be distinct from detrimental mechanisms that reduce the magnitude of baroreflex-mediated changes in SNA and HR only in the male OZRs.

Several observations strongly suggest that the development of unremitting hyperglycemia is a catalyst for the impaired baroreflex efficacy seen in the young adult male OZRs. First, diminished baroreflexes occur with chronic hyperglycemia in the absence of other defining traits of MetS. Rats and humans with type 1 diabetes have diminished baroreflex control of HR without obesity, hyperinsulinemia, or hypertension (50, 51). Likewise, in male and female fructose-fed rats, hyperglycemia is associated with diminished baroreflex efficacy in the absence of obesity (52), and fructose-fed rats are not hypertensive when AP is measured by telemetry (53). Chronic hyperglycemia abolishes the ability of glucose to enhance release of glutamate from afferent terminals in the NTS (54), and type 1 diabetic rats and insulin-resistant, hyperglycemic male OZRs develop diminished baroreflex-mediated activation of the NTS (38, 55). Second, restoration of glycemic control in fructose-fed rats or male OZRs improves baroreflex-mediated bradycardia (38, 52). Similarly, restoration of glycemic control enhances baroreflex-mediated activation of the NTS in male OZRs (38). Third, female OZRs and fructose-fed female rats are resistant to the development of chronic hyperglycemia (Fig. 7) (56), coincident with delayed development of impaired baroreflexes (36). In the present study, young adult female OZRs had hypertension, hyperinsulinemia, and dyslipidemia compared with female LZRs, but the magnitude of baroreflex-mediated bradycardia and activation of the NTS were comparable to LZRs coincident with absent or mild hyperglycemia. Together, these observations strongly suggest that unlike males, females are protected from developing the pronounced chronic hyperglycemia caused by excess ingestion of fat, fructose, or total calories. Furthermore, because young adult females with obesity and insulin resistance maintain normal blood glucose levels despite excess ingestion of calories and weight gain, they retain robust baroreflex-mediated activation of the NTS and baroreflex efficacy.

In the present study, estrogen could contribute to the preserved baroreflex efficacy directly or by supporting glucose homeostasis in female OZRs. Numerous actions of estrogen in the periphery have the capacity to boost glycemic control, including enhancement of insulin signaling in insulin-sensitive tissues, release of insulin from the pancreas, adipose tissue metabolism and distribution pattern, and reduced hepatic glucose production (28, 30, 57). In agreement, compared with males, cycling females have better glycemic control that is lost after menopause or ovariectomy (58–60). In addition, circulating and central estrogen also enhance baroreflex efficacy independent of actions upon glucose homeostasis (61–63). Estrogen increases the density of cardiac cholinergic muscarinic receptors (64), and local injections of estrogen directly into the NTS enhance baroreflex-mediated bradycardia (63). In agreement, baroreflex gain fluctuates during the estrous cycle, with baroreflex gain and upper plateau being greatest during phases with higher circulating estrogen (45). Baroreflex gain is reduced after ovariectomy (61), and peripheral administration of exogenous estrogen in ovariectomized rats enhances baroreflex efficacy (61, 65). However, the lower plateau of the baroreflex is not affected by phase of the estrous cycle (46), and the most prominent baroreflex deficit in OZRs is the inability to maximally inhibit SNA and HR by raising AP. Thus, estrogen is more likely to preserve baroreflex efficacy by maintaining normal blood glucose in female OZRs, rather than a direct role in baroreflex-mediated activation of the NTS. Furthermore, although estrogen is a likely contributor to differences in baroreflex efficacy in young adult male and female OZRs, given the disparity in glycemic control, estrogen is not likely to explain the difference in baroreflex efficacy in 6-mo-old female OZRs and LZRs. Further study will be necessary to elucidate roles of estrogen for differences in baroreflex efficacy in Zucker rats.

Mechanisms underlying the development of impaired baroreflex bradycardia in 26- to 27-wk-old female OZRs are not readily apparent. Estrogen should still be present and likely contributes to their sustained glycemic control at this age. Fed glucose levels in 24 to 27-wk-old female OZRs were similar to fed glucose in male OZRs treated with metformin or pioglitazone, and these treatments restore baroreflex-mediated bradycardia and activation of the NTS in male OZRs (38). Comparable PE-induced c-Fos expression in the NTS of 26- to 27-wk-old female OZRs and LZRs suggests baroreflexes are not impaired by the same mechanisms seen in younger male OZRs (33, 38). Because sympathetic baroreflexes were not evaluated in rats at this age, it is unclear whether reduced baroreflex bradycardia is caused by altered efferent nerve activity or response of the heart to parasympathetic inputs. In support of diminished influence of parasympathetic efferent nerves upon HR, stimulation of a vagal efferent nerve evokes smaller decreases in HR in 12-mo-old female rats made obese by a high-fat diet (66). Interestingly, in these lean and obese rats, raising AP by PE infusion or direct stimulation of aortic depressor nerve evokes comparable decreases in SNA (66). These data suggest that impaired baroreflex bradycardia is isolated to parasympathetic actions at the heart in rats with obesity, similar to observations of impaired cardiac but not sympathetic baroreflexes in normotensive humans with obesity (12, 66). Further progression of other MetS traits could also promote diminished baroreflex efficacy in female OZRs. For instance, plasma lipids continue to rise in female OZRs, and increased liver fat content is strongly associated with reduced cardiac vagal tone and efficacy of baroreflex bradycardia (16). Future studies will be necessary to determine whether other MetS traits contribute to impaired baroreflex bradycardia in 26- to 27-wk-old female OZRs and whether these changes are specific to females with obesity.

### Perspectives and Summary

Sex differences in the onset and severity of MetS traits provide valuable clues for underlying mechanisms that could facilitate prevention or reversal of harmful consequences of obesity. Although MetS traits are often assessed and treated separately, their comorbid presentation suggests that consideration of interactions is essential for optimal treatment (17). Furthermore, some deleterious traits, such as impaired baroreflexes, are not routinely measured or treated, leaving patients with obesity at risk for adverse outcomes (9). In the absence of obesity and hypertension, diminished baroreflexes are independently associated with insulin resistance, suggesting a causative relationship between impaired glycemic control and baroreflex efficacy (15). Like humans with obesity, OZRs and rodents made obese by a high-fat diet to develop diminished baroreflexes that can occur with hypertension, dyslipidemia, and insulin resistance (11, 19, 30, 38, 49, 66). In male OZRs, hyperglycemia appears to foster impaired activation of NTS neurons that tonically inhibit SNA to cardiovascular targets (38). This state diminishes sympathoinhibitory reflexes, such as the baroreflex, and reduces restraint on sympathetic vasomotor tone (5, 43). As seen in women with obesity, female OZRs and rodents made obese by a high-fat diet to maintain glycemic control in the presence of a higher percentage weight gain, hyperinsulinemia, and dyslipidemia compared with males (19, 30). The present study showed that preserved glucose homeostasis in young adult female OZRs coincided with maintaining the ability increased AP to activate the NTS to reduce SNA and HR and generate baroreflexes. Although future studies are needed to clarify cellular mechanisms underlying hyperglycemia-induced changes in barosensitive NTS neurons in male OZRs and how female OZRs maintain glucose homeostasis, these data provide insights into important sex differences. Interestingly, the development of impaired baroreflex efficacy in older female OZRs occurred without profound hyperglycemia or diminished NTS activation. Thus, later development of diminished baroreflexes in female OZRs was not simply a delayed onset of the same deficit seen in male OZRs. These data highlight a significant limitation of examining males to understand the onset and progression of MetS traits in females with obesity and their underlying mechanisms for impaired autonomic regulation of AP.

## GRANTS

This project was financially supported by National Heart, Lung, and Blood Institute Grant R01-HL-132568 (to A. M. Schreihofer). P. Chaudhary was supported by predoctoral fellowship PRE27260088 from the American Heart Association and a training grant from the National Institutes of Health Grant T32 HL07224.

## DISCLOSURES

No conflicts of interest, financial or otherwise, are declared by the authors.

## AUTHOR CONTRIBUTIONS

P.C., P.D.-E., and A.M.S. conceived and designed research; P.C., P.D.-E., and A.M.S. performed experiments; P.C., P.D.-E., and A.M.S. analyzed data; P.C., P.D.-E., and A.M.S. interpreted results of experiments; P.C., P.D.-E., and A.M.S. prepared figures; P.C., P.D.-E., and A.M.S. drafted manuscript; P.C., P.D.-E., and A.M.S. edited and revised manuscript; P.C., P.D.-E., and A.M.S. approved final version of manuscript.
